# Female assortative mate choice functionally validates synthesized male odours of evolving stickleback river–lake ecotypes

**DOI:** 10.1098/rsbl.2018.0730

**Published:** 2018-12-12

**Authors:** Christoph L. Gahr, Thomas Boehm, Manfred Milinski

**Affiliations:** 1 Max Planck Institute for Evolutionary Biology, August-Thienemann-Strasse 2, 24306 Plön, Germany; 2 Department of Developmental Immunology, Max-Planck-Institute for Immunobiology and Epigenetics, Stübeweg 51, 79108 Freiburg, Germany

**Keywords:** ecological speciation, major histocompatibility complex, olfaction, mate choice, habitat-specific signal, synthesized odour

## Abstract

During mate choice decisions, females of many vertebrates use male olfactory cues to achieve immunogenetic optimality of their offspring. Three-spined sticklebacks (*Gasterosteus aculeatus*) populating habitats that differ in their parasite communities evolve locally adapted combinations of genetic variants encoded at the major histocompatibility complex (MHC). Such adaptation confers optimal resistance to the local parasite fauna. Immunogenetic signatures co-evolved with local parasites favour population-specific assortative mate choice behaviour. Previous studies have shown that female sticklebacks evaluate male MHC-associated olfactory cues during the process of mate choice, but how habitat-specific information is exchanged between males and females has remained elusive. Here, we directly demonstrate the molecular nature of the olfactory cue providing habitat-specific information. Under controlled laboratory conditions, females that are ready to mate prefer mixtures of synthetic MHC peptide ligands mimicking the optimal allele number of their original population. These results imply that female sticklebacks can determine the number of MHC alleles of their prospective mates, compare it to their own immunogenetic status, and, if optimal with respect to the immunogenetic complementarity, accept the male as mate. Our results suggest a potentially common mechanism of ecological speciation in vertebrates that is based on the olfactory assessment of habitat-specific immunogenetic diversity.

## 1. Introduction

Eco-evolutionary dynamics shape the genotypic and phenotypic characteristics of populations and individuals. Since the survival of populations ultimately depends on the maintenance of genotypes optimally adapted to the specific habitat, mate choice is critical for producing high-quality offspring [1]. Of particular value for mate choice decisions are physiologically costly signals, such as bright colours [2] or elaborate bird songs [3], because they honestly reveal physical health and thus potential resistance against prevailing parasites [4].

**Figure 1.**
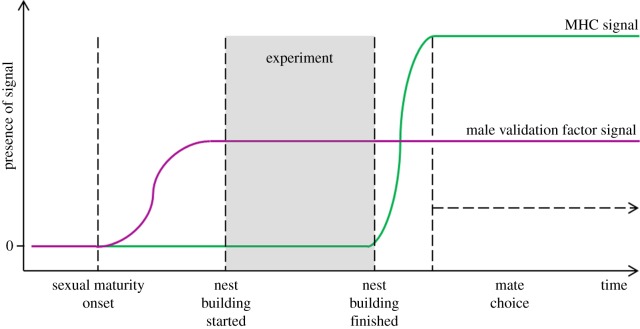
Presence of major histocompatibility complex (MHC) signal and male validation factor signal during the breeding cycle (adapted from [6,7]). Only males that produced the male validation factor but not yet the MHC signal were used.

Sexually mature three-spined stickleback males (*Gasterosteus aculeatus*) seek out and defend a territory, build a nest out of plant material and create a scent trail to attract females. Apart from the breeding coloration, the most intriguing signal assessed by choosing females is the odour signal created by the male [5]. The odour contains two major components (figure 1): a so-called male validation factor [6–8] signals the presence of a male ‘stickleback’ and thus validates the second odour component, which is associated with an individual male's major histocompatibility complex (MHC) profile [5,9]. MHC molecules inform the immune system of the composition of intracellular proteins and thus are critical mediators of an immune response that is initiated when a cell contains foreign protein(s), for instance, after an infection [10]. MHC genes are the most polymorphic genes in vertebrates and their high allelic diversity—which is maintained by both parasite [11] and sexual selection [2,4]—is advantageous when dealing with complex parasite communities and changing immunological challenges [12–14].

MHC molecules bind different subsets of intracellular peptides (including those that are produced by pathogens) and present them at the cell surface to the antigen receptors of T cells. The greater the number of structurally distinct MHC molecules an individual possesses, the greater the sequence space of intracellular proteins that becomes detectable by the immune system. However, because the repertoire of T cell receptors must be purged from overly self-reactive versions to avoid inadvertent self-destruction, a trade-off exists between the ability to recognize foreign antigens and the avoidance of autoimmunity; as a result, each individual expresses only a small subset of MHC alleles present in the population [15,16]. This optimal number of MHC alleles varies among populations exposed to different parasite faunas but is considered to be an ecotype-specific parameter of local adaptation: the optimum increases with the number of parasite species in a population [8,17,18].

Sensory evaluation of MHC genotype focuses on anchor amino acids of the peptide ligands, which are structural mirror images of the genetically encoded binding grooves of the MHC molecules. MHC peptide ligands are liberated from the peptide–MHC complexes and appear in bodily fluids; in this way, they become available for olfactory assessment. Studies in mice [19], fish [20] and humans [21] suggest that MHC peptides are widely employed as olfactory cues that influence vertebrate social behaviour including mate choice. Indeed, supplementing water from male sticklebacks' nests with synthetic versions of MHC-ligand peptides altered the male's perceived MHC profile and predictively changed female choice [20].

During mate choice decisions, female sticklebacks choose males optimally complementing their own set of MHC alleles in such a way that the offspring are close to the population-specific optimal number [8]. A direct link between non-random mating and functional relevance of habitat-adapted alleles has been dubbed ‘magic trait’ by Gavrilets [22] and can be seen as a powerful accelerator of ecological speciation [23]. However, although our previous experiments based on the outcome of interference with natural odours strongly suggested the possibility that MHC peptide ligands function as a mechanistic underpinning of MHC-associated behaviour, this hypothesis has remained so far untested. Here, using sticklebacks from two physically connected populations that are ecologically separated by different parasite faunas, we identify the molecular nature of the population-specific MHC-associated odour signal.

## 2. Material and methods

### (a) Animal origin and housing

Wild-caught three-spined sticklebacks originated from a lake and a connected river in northern Germany. The fish were caught in December 2017 and cycled through winter (6°C, 12 L : 12 D), spring (12°C, 12 L : 12 D) and finally summer (18°C 18 L : 6 D) conditions in the laboratory. Fish were housed individually until the experiments. Males were provided with standardized nesting material consisting of green polyester threads. Nest [24] progression was monitored daily. Male sticklebacks will not produce the MHC signal until their nest is finished, when they start ‘creeping through the nest’ [6]. However, the male validation factor is present from the onset of nest building ([6], figure 1); this offers the possibility to expose females to the male validation factor without the natural male-derived MHC component of the signal peptides.

All animal experiments described were approved by the Ministry of Nature, Environment and Country Development, Schleswig Holstein, Germany (project number: 1096).

### (b) Experimental design

Gravid female sticklebacks were placed in a flow chamber fed by two columns with laminar water flow [5,9] and video-recorded when choosing between the two front quarters of the chamber for two periods of 300 s each (= 600 s total), with spatial reversal of the water source after the first 300 s period. The remaining space was regarded as neutral and not scored. Determining odour preference in this set-up has been shown to reliably predict mate choice (see supporting text of [20]), which allows for testing the effect of synthetic peptides on female preference. To this end, for each run, we took two 1 litre water samples from the tank of a single male (containing male validation factor but no MHC-associated component ([6]; see above)) and spiked each of these with either 2 or 4 synthetic peptides as described [20]. As each female was presented with water from two different males in two separate runs, and each male was used twice with two different females, we sampled the male tank twice, once for each run. When used as river-like stimulus, two peptides in solvent were continuously added to one half of the flow channel; when used as a lake-like stimulus, four peptides in solvent were continuously added to the other half of the flow channel. Each female was tested using water taken from the tank of a sympatric and an allopatric male, within a 1 h interval. All experiments were performed in double-blinded fashion in the Plön laboratory. Each female–male combination was used only once to avoid pseudo replication and thus is a single independent statistical unit.

### (c) MHC analysis

DNA was extracted from clipped spines using the DNeasy 96 Blood & Tissue Kit (Qiagen). MHC allele numbers were measured using Reference Strand-mediated Conformation Analysis (RSCA) as described [25].

### (d) Peptides

The four different MHC-ligand peptides used in this study were: SYIPSAEKI, SFVDTRTLL, ASNENMETM and AAPDNRETF [6,20]. Peptides were chemically synthesized, purified, verified by mass spectroscopy (MALDI-TOF) and dissolved in phosphate-buffered saline (PBS), as described [20].

### (e) Statistical analysis

All statistical analyses were done in RStudio (v. 1.0.136) (see http://www.rstudio.com/).

## 3. Results

**Figure 2.**
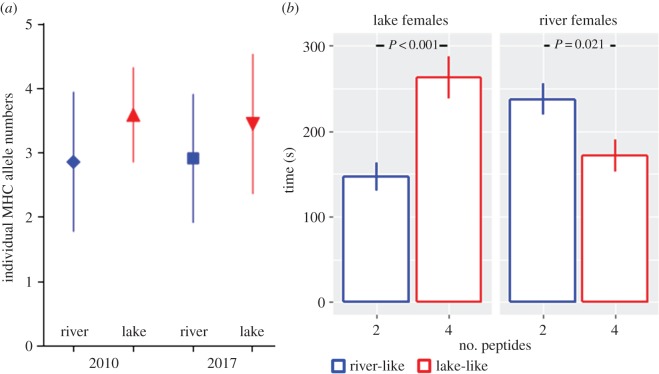
(*a*) Mean (±s.e.) MHC allele numbers from the wild-caught sticklebacks from river and lake populations. Despite being caught seven years apart, the wild population averages remain almost identical between the fish from this study (caught in 2017) and the ones used by Andreou *et al*. [8], caught in 2010. (*b*) Habitat-specific female preference for synthesized male MHC profiles. Time (s) (mean ± s.e.) of 600 s the female spent in the quarter of the test chamber where either two or four peptides arrived with the current.

We first determined the population-specific optima of MHC class IIB allele numbers in wild-caught fish of lake (Großer Plöner See (GPS); *n* = 53 fish) and river (Söhrener Au (SAU); *n* = 49 fish) populations sampled in 2017. Consistent with previous work, we find that the MHC optimum is higher in lake (3.453 ± 1.084, mean ± s.e.) than in river (2.918 ± 0.997, River2017–Lake2017, *p* = 0.011, *t* = −2.588, df = 100, two-tailed *t*-test) fish. Remarkably, the population-specific MHC optima determined in 2010 (lake: 3.595 **±** 0.735, *n* = 42; river: 2.865 ± 1.084, *n* = 37, River2010–Lake2010: *p* = 0.0007, *t* = 3.54, df = 77) do not differ significantly from those from 2017 (difference between River2017 and River2010: *p* = 0.85, *t* = 0.2351, df = 84; Lake2017–Lake2010: *p* = 0.47, *t* = 0.7266, df = 93), indicating immunogenetic stability of these two populations (figure 2*a*). Previous findings showed that the mean individual number of MHC alleles [5] corresponds with the local optimum [14].

Mice [19] and fish [20] decode MHC allelic complexity by olfactory evaluation of the typically two anchor residues of the peptide ligands after they have become liberated from the binding pockets of the MHC molecules [26], whereas the other residues in the peptides are of minor importance [19]. For sticklebacks, exposure to conditioned water in a flow chamber reliably predicts mate choice (see the supplementary information for ref. [20]). In order to mimic the habitat-specific number of MHC alleles in a mate choice experiment, we exploited the observation that lake sticklebacks rarely exhibit only two alleles or fewer, and, correspondingly, that river sticklebacks rarely carry four or more alleles (figure 2*a*). Therefore, when a female is confronted with a male odour containing only two different kinds of peptides, she would be rarely mistaken in assuming that this signal comes from an individual of the river ecotype; an odour with three peptides would be ambiguous in origin, whereas an odour containing four peptides would most of the time come from an individual of the lake ecotype. Hence, our synthetic river-specific and lake-specific MHC mimics consisted of two and four peptides, respectively.

In the next set of experiments, we confirmed that the allele-specific combination of anchor residues is the functionally relevant property of the MHC peptide mimics. The comparatively small number of MHC alleles per individual ensures that the discriminatory power of the olfactory system is rarely challenged. According to the two-anchor site rule of ligand binding, one anchor pocket can accommodate one out of the 20 amino acids, resulting in a maximum of (20 × 20 =) 400 decodable alleles. This proved to be the case. For the two-peptide signal, two random exclusive combinations from the four peptides were created; the outcome of choices by both river and lake females tested with one or the other two-peptide combination did not differ significantly (*n* = 45, *t*_29_ = 1.2402, *p* = 0.23; two-tailed *t*-test).

When females of the lake ecotype were confronted with a choice of four (the ‘lake mimic’) or two (the ‘river mimic’) peptide cocktails in the flow chamber experiment, lake females spent significantly more time in front of the four-peptide inlet compared to the one with two peptides (figure 2*b*; *n* = 12, *t*_18_ = −3.9659, *p* = 0.00091, two-tailed paired *t*-test). The river females, by contrast, significantly preferred the two-peptide signal (figure 2*b*; *n* = 14, *t*_25_ = 2.4822, *p* = 0.021). Females thus showed a significant preference for their habitat-specific synthetic MHC allele number. Control experiments corroborated that the male validation factor, which is required for accurate interpretation of the MHC-based signal by female sticklebacks [6], is invariant between lake and river males [8]. Indeed, the preference for either two-peptide or four-peptide mimics was independent of the male's population of origin, both for the river (*n* = 12, *t*_22_ = −1.0063, *p* = 0.33) or the lake (*n* = 12, *t*_21_ = −0.16543, *p* = 0.87), i.e. when the male providing the validation factor was either a river or a lake male for both the two- and the four-peptide inlet.

## 4. Discussion

The sexual selection strategy used by sticklebacks incorporates an odour-based assessment of MHC diversity [5,9]. Here we have shown that a habitat-specific male odour signal that determines the outcome of assortative mate choice can be reduced to synthetic peptide combinations, whose anchor sequence diversities reflect the numbers of MHC alleles possessed by sticklebacks of a typical river ecotype or of a typical lake ecotype.

The exceptional diversity of MHC alleles in a population and their defining combinations of anchor residues explain why during the olfactory assessment of MHC diversity individual female sticklebacks count each synthetic peptide ligand as representing one allele [20], if accompanied by the stickleback validation factor. During mate choice, female sticklebacks need to distinguish between the number and the quality of MHC alleles, whereas their quality in terms of their immunoprotective capacity becomes important during the second part of mate choice [9]. After a female has approached a male that offers the habitat-specific optimal number of MHC alleles, she spawns only if the male is brightly red [2], indicating that the male's alleles also confer resistance against the current parasite fauna.

The parasite faunas of lakes and rivers are largely distinct, with lakes exhibiting a greater diversity of parasites [18,27], reflected in a greater number of MHC IIB alleles in populations of lake sticklebacks [8,18]; correspondingly, river sticklebacks carry fewer MHC IIB alleles. Ecological speciation has set in to separate the two ecotypes through pre-zygotic mating barriers, as a consequence of the fact that lakes and rivers are connected with each other [18]. During mate choice, female sticklebacks evaluate MHC diversity of prospective males in order to be able to maintain the different optima in the number of individual MHC alleles [14,17]. Because MHC alleles of lake fish provide resistance to lake parasites, and the river MHC composition to river parasites [28], the evaluation of their MHC genes possesses the quality of a magic trait that links habitat-specific adaptation with assortative mate choice [22,23].

Because different natural habitats can be assumed to harbour different parasite communities, mate choice employing olfactory assessment of habitat-specific immunogenetic diversity may represent a common mechanism of ecological speciation in vertebrates.

## Supplementary Material

Extended Material & Methods

## Data accessibility

Data available from the Dryad Digital Repository: http://dx.doi.org/10.5061/dryad.16qk7vf [29].
