# Supplementary material for: Female assortative mate choice functionally validates synthesized male odours of evolving stickleback river–lake ecotypes
Source: Biol Lett. 2018 Dec 12;14(12):20180730. doi: 10.1098/rsbl.2018.0730 (PMC6303515; doi:10.1098/rsbl.2018.0730)
Supplement: Extended Material & Methods [file rsbl20180730supp1.docx]

EXTENDED METHODS FOR SI:

**Materials and Methods**
*Animal origin and housing*.
All fish used in this study were wild-caught three-spined sticklebacks (*Gasterosteus aculeatus*) originating from the Grosser Plöner See lake (n = 53, 54˚14’61.0’’N, 10˚40’86.9’’E) and the Sörener Au river (n = 53, 54˚22’50.1’’N, 10˚60’91.5’’E) in northern Germany. The fish were caught in December 2017 and cycled through winter (6˚C, 12:12 L:D), spring (12 ˚C, 12:12 L:D) and finally summer (18 ˚C 18:6 L:D) conditions in the laboratory. Fish were housed individually upon transfer to the summer conditions, fed *ad libidum* with live *Chironomidae spec.* larvae and were spine-clipped for sex-typing and MHC-allele analysis.

Males were provided with standardized nesting material consisting of green polyester threads (cut to a length of ~10cm), half a petri dish half-filled with sand and a small rock. Nest progression was monitored daily and nest status was determined based on appearance and male behavior (see 24 for details), using only males with unfinished nest for the experiment.
Male sticklebacks will not produce the MHC signal until their nest is finished when they start ‘fanning’ and ‘creeping through the nest’ (Fig. 1), suggesting that it is costly for the male to produce the MHC-associated signal and/or to attract females to the nest too early (6). As MHC molecules are shed from the cell surface (Singh et al., 1987), it is assumed that this process changes the conformation of the peptide binding groove, causing faster liberation of peptides which then become available for assessment via other sensory modalities, such as the vomeronasal system (Leinders-Zufall et al., 2009). However, the male validation factor is present from the onset of nest building (6, Fig. 1); this offers the possibility to expose females to the male validation factor without the natural male-derived MHC component of the signal peptides (6, Fig.1).

All animal experiments described were approved by the Ministry of Nature, Environment and Country Development, Schleswig Holstein, Germany.

*Experimental design.*

Gravid female sticklebacks were placed in a flow chamber fed by two columns with laminar water flow (5, 9). Females were able to freely investigate the water composition in the chamber for two periods of 300s each, with spatial reversal of the water source after the first 300s period. Determining odor preference in this setup has been shown to reliably predict mate choice (ref. 19, *supporting text*), which provides the opportunity to test the effect of synthetic peptides on female preference. To this end, water was taken from the tank of a single male (containing male validation factor but no MHC-associated component (6; see above) per trial and converted to fully functional stimulus water by addition of synthetic peptides. When used as river-like stimulus, two peptides in solvent were continuously added to one half; when used as a lake-like stimulus, four peptides in solvent were continuously added to the other half of the flow channel. The concentration of peptides in solvent and the volume of supplement added to the water columns were identical on both sides in all experiments. Each female was tested using water taken from the tank of a sympatric and an allopatric male, within a one-hour interval. All experiments were performed in double-blinded fashion in the Plön laboratory. Each female-male combination was used only once to avoid pseudo replication and thus is a single independent statistical unit. Each fish was only used once with the exception of two males that were allowed to build a new nest and then tested again with two different females.

*Run validation.*
To validate a female’s principal readiness to spawn during the trial, she had to spontaneously spawn in her home tank in the absence of a male within 24h after her second test (20). Further, females that remained on the same side in the 1st and 2nd run of a trial and thus revealed an unwillingness of exploration were designated as ‘no choice’ and excluded. Three runs were excluded because one of the two males showed clear fanning behavior in their home tank between tests indicating the start of producing the MHC signal on the trial day; these males might have produced the MHC signal already before the start of the trial.

*MHC-analysis.*
DNA was extracted from clipped spines using the DNeasy 96 Blood & Tissue Kit (Qiagen), following the manufacturer´s protocol. MHC allele numbers were measured using Reference Strand-mediated Conformation Analysis (RSCA) as described (25). The resulting measurements were analysed using the GeneMarker software (Version 2.4.2, Softgenetics).

*Peptides.*
The four different MHC-ligand peptides used in this study were: SYIPSAEKI, SFVDTRTLL, ASNENMETM, and AAPDNRETF (20, 6). Peptides were chemically synthesized, purified, verified by mass spectroscopy (MALDI-TOF), and dissolved in phosphate-buffered saline (PBS), as described (20). Two or four peptides were chosen as to represent two distinct allele combinations mimicking either a definite river or a definite lake male MHC genotype; as can be seen from Fig. 2a, it is extremely unlikely that a river male has 4 alleles and that a lake male has 2 alleles. For the 2-peptide signal, two random exclusive combinations from the four peptides were created. Females tested with one (SYIPSAEKI and SFVDTRTLL**,** n=32) and females tested with the other (ASNENMETM and AAPDNRETF**,** n=13) combination did not differ significantly in their choice (n=45, t(29) =1.2402, P=0.2247, two-tailed t-test). The discrepancy between the “n” for each two-peptide combination is due to unsuccessful runs (e.g. female did not spawn within 24h after the final run) which happened to be more common in the 2^nd^ (ii) combination and to fewer ripe females available towards the end. Females did however not
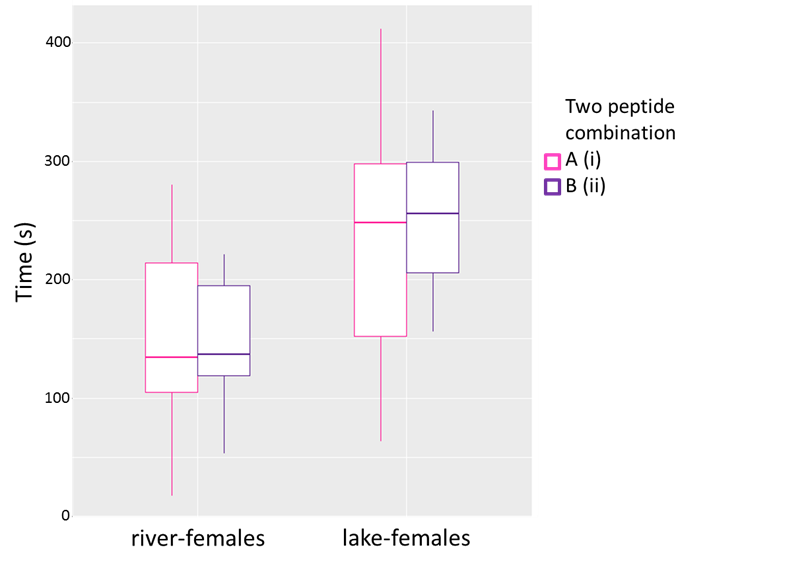
differentiate between either of the two peptide combinations (P=0.7481, for river females and P=0.9024 for lake females, Mann-Whitney U-test, two-tailed (see Figure Si1).

**Figure Si1:** Both river and lake females show no significant preference for either of the two random two peptide combinations used in the study. Time (s) (median + first and second quartiles) of 600 s the female spent in the quarter of the test chamber where the two-peptide combination arrived.

*Statistical analysis.*
All statistical analysis were done in RStudio (version 1.0.136) using the build in packages for statistical analysis (two-tailed t-test) and the ggplot2 package for graphical representation. Times from the 1st and 2nd run of each female-male combination were added up and regarded as one variable (controlling for weak side preference). The data did not significantly differ from a normal distribution (Shapiro-Wilk, P=0.384), hence allowing the use of parametric tests for data analysis.

**References:**

Singh PB, Brown RE, Roser B 1987 MHC antigens in urine as olfactory recognition cues. *Nature* **327**:161-164. doi: 10.1038/327161a0

Leinders-Zufall T, Ishii T, Mombaerts P, Zufall F, Boehm T 2009 Structural requirements for the activation of vomeronasal sensory neurons by MHC peptides. *Nat. Neurosci.* **12**,1551-1558. doi: 10.1038/nn.2452
